# Ginsenoside Rg1 Ameliorates the Learning and Memory Deficits of 5xFAD Mice by Inhibiting CCR3 Activity: Insights from In Vivo and In Vitro Investigations

**DOI:** 10.3390/ph19050661

**Published:** 2026-04-23

**Authors:** Hui Lu, Ying Yu, Ying Yang, He Li, Yangyi Li, Tianhao Yu, Shixue Wang, Fengzhen Li, Xiaorui Cheng

**Affiliations:** Institute Innovation of Chinese Medicine and Pharmacy, Shandong University of Traditional Chinese Medicine, Jinan 250355, China; aloe58484@163.com (H.L.); 13969179693@163.com (Y.Y.); qdrdyangying@163.com (Y.Y.); 17853737527@163.com (H.L.); liyangyie@163.com (Y.L.); ythjason@163.com (T.Y.); wangsx1226@126.com (S.W.); wslfz5521@163.com (F.L.)

**Keywords:** Alzheimer’s disease, CCR3, Ginsenoside Rg1, neuroprotection

## Abstract

**Background/Objectives**: Alzheimer’s disease (AD) is characterized by amyloid-beta accumulation and neuroinflammation, yet the molecular target of Ginsenoside Rg1 remains elusive. This study aimed to elucidate the neuroprotective mechanism of Ginsenoside Rg1, specifically investigating its interaction with C-C motif chemokine receptor 3 (CCR3). **Methods**: We utilized 5xFAD transgenic mice and CCR3-overexpressing BV2 microglial cells. Behavioral assessments, enzyme-linked immunosorbent assays, quantitative real-time polymerase chain reaction, molecular docking, and surface plasmon resonance were employed to evaluate cognitive function and molecular pathways. **Results**: Ginsenoside Rg1 treatment significantly ameliorated spatial learning and memory deficits. Quantitatively, Rg1 reduced cortical amyloid-beta 1–40 levels (*p* < 0.05) and bound directly to CCR3 with a dissociation constant of 3.599 × 10^−5^ mol/L. This inhibition suppressed neuroinflammation and restored neurotrophic factors, including Brain-derived neurotrophic factor. **Conclusions**: CCR3 is a novel pharmacological target for Ginsenoside Rg1, providing a precise molecular basis for its neuroprotective effects. Future research should focus on clarifying the pharmacokinetic profile and brain bioavailability of Ginsenoside Rg1 to facilitate clinical translation.

## 1. Introduction

Alzheimer’s disease (AD) represents one of the most formidable public health challenges of the 21st century. As global populations age, the prevalence of AD has reached epidemic proportions, imposing a staggering socioeconomic burden on healthcare systems and families worldwide [1,2,3,4]. Despite extensive research, current clinical interventions—primarily acetylcholinesterase inhibitors—offer only symptomatic relief rather than disease modification [5]. Consequently, there is an urgent, unmet clinical need to identify novel therapeutic targets capable of halting or reversing the underlying neurodegenerative processes, which are primarily driven by amyloid-beta (Aβ) pathology and chronic neuroinflammation [6,7].

Neuroinflammation is a hallmark of AD pathology, driven by complex interactions between the central nervous system and peripheral immune responses. C-C motif chemokine receptor 3 (CCR3), a receptor critical for immune cell migration, has emerged as a key player in this context [8]. CCR3 is expressed not only in neurons but also in peripheral immune cells, such as eosinophils (EOSs) [9], which are increasingly recognized for their role in inflammatory diseases [10,11,12]. Previous studies have demonstrated that CCR3 antagonism or knockdown can mitigate cognitive impairment and hippocampal dysfunction by suppressing proinflammatory cytokine production [13,14,15]. Furthermore, the CCR3 ligand C-C motif chemokine ligand 11 (CCL11) has been implicated in age-associated cognitive decline and hippocampal impairment [16,17,18]. Other CCR3 ligands, including C-C motif chemokine ligand 24 [19], C-C motif chemokine ligand 2 [20,21] and C-C motif chemokine ligand 5 (CCL5) [22], further contribute to the neuroinflammatory environment characteristic of AD.

Ginsenoside Rg1 (Rg1), a primary bioactive component derived from *Panax ginseng*, has garnered significant attention for its neuroprotective potential [23]. Extensive preclinical evidence indicates that Rg1 ameliorates cognitive deficits across diverse AD animal models [24], including those induced by type 2 diabetes [24], alcohol exposure [25], and D-galactose [26] or lipopolysaccharide administration [27]. Mechanistic studies have shown that Rg1 reduces inflammatory protein expression, such as NLR family pyrin domain-containing 1 and caspase-1, in Amyloid precursor protein/Presenilin 1 (APP/PS1) transgenic mice [28,29]. Pharmacokinetic data further suggest that Rg1 can cross the blood–brain barrier, albeit with rapid elimination [30]. Despite these findings, the precise molecular mechanisms underlying its therapeutic action remain unclear.

To bridge this knowledge gap, this study investigates the potential interaction between Rg1 and CCR3 signaling. We hypothesize that Rg1 exerts its neuroprotective effects by inhibiting CCR3 activity, thereby attenuating neuroinflammation and reducing Aβ pathology in AD.

## 2. Results

### 2.1. Treatment with Rg1 Ameliorates Cognitive Impairment in 5xFAD Mice

**Figure 1:**
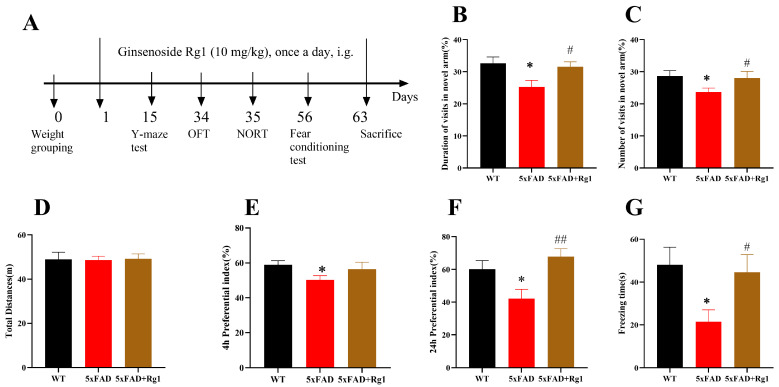
Treatment with Rg1 ameliorated cognitive impairment in 5xFAD mice. (**A**) Schematic diagram. (**B**) Percentage of time spent in novel arm and (**C**) percentage of entries into novel arm in Y-maze test. (**D**) Total distance traveled in OFT. (**E**) PI at 4 and (**F**) 24 h after learning period in NORT. (**G**) Freezing time in fear conditioning test. Mean ± SEM; *n* = 8. * *p* < 0.05, vs. WT, Student’s *t*-test; ## *p* < 0.01, # *p* < 0.05, vs. 5xFAD, Student’s *t*-test.

Cognitive performance in 5xFAD mice was evaluated using a behavioral battery, including the Y-maze test, open field test (OFT), novel object recognition test (NORT) and fear conditioning test (Figure 1A). In the Y-maze, 5xFAD mice displayed significant cognitive impairment, evidenced by a marked reduction in both the duration spent in the novel arm (Figure 1B; *p* < 0.05) and the percentage of entries into that arm (Figure 1C; *p* < 0.05). Conversely, Rg1 treatment effectively rescued these deficits, as indicated by a significantly increased duration in the novel arm (Figure 1B; *p* < 0.05) and a higher percentage of entries into the novel arm (Figure 1C; *p* < 0.05).

Statistical analysis revealed no significant differences in the total distance traveled among the three groups of the OFT (Figure 1D). After 4 and 24 h of learning in the NORT (Figure 1E,F; *p* < 0.05), the Preference index (PI) was significantly lower in 5xFAD mice than in Wild-type C57BL/6J (WT) mice, whereas the administration of Rg1 resulted in a significant increase in the PI after 24 h of learning (Figure 1F; *p* < 0.01). Freezing time was decreased in 5xFAD mice (Figure 1G; *p* < 0.05) in the fear conditioning test. Additionally, the administration of Rg1 significantly increased the freezing time of 5xFAD mice (Figure 1G; *p* < 0.05). The aforementioned data indicate that treatment with Rg1 ameliorated cognitive impairment in 5xFAD mice, including improvements in spatial learning and memory, long-term object recognition memory and fear memory.

### 2.2. Treatment with Rg1 Reduces the Aβ Content in the Cortex of 5xFAD Mice

**Figure 2:**
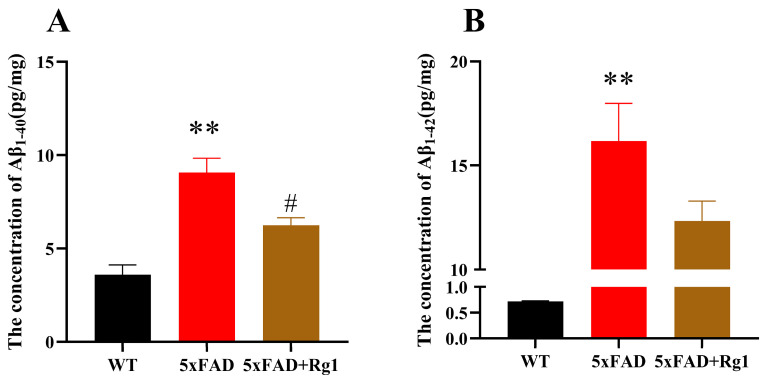
Rg1 reduced cortical Aβ content in 5xFAD mice. The levels of (**A**) Aβ_1–40_ and (**B**) Aβ_1–42_ in the cortex were detected via ELISA. Mean ± SEM; *n* = 8. ** *p* < 0.01, vs. WT, Student’s *t*-test; # *p* < 0.05, vs. 5xFAD, Student’s *t*-test.

The administration of Rg1 led to significantly lower levels of Aβ_1–40_ (Figure 2A; *p* < 0.05) in the cortex of 5xFAD mice compared with C57 mice. Although a decreasing trend was observed in Aβ_1–42_ levels, the difference was not statistically significant (Figure 2B). Rg1 treatment partially alleviated amyloid pathology, primarily by modulating Aβ_1–40_ levels in 5xFAD mice, as evidenced by the aforementioned results.

### 2.3. Treatment with Rg1 Decreases the Levels of CCR3 and CCL11 in the Brains of 5xFAD Mice

**Figure 3:**
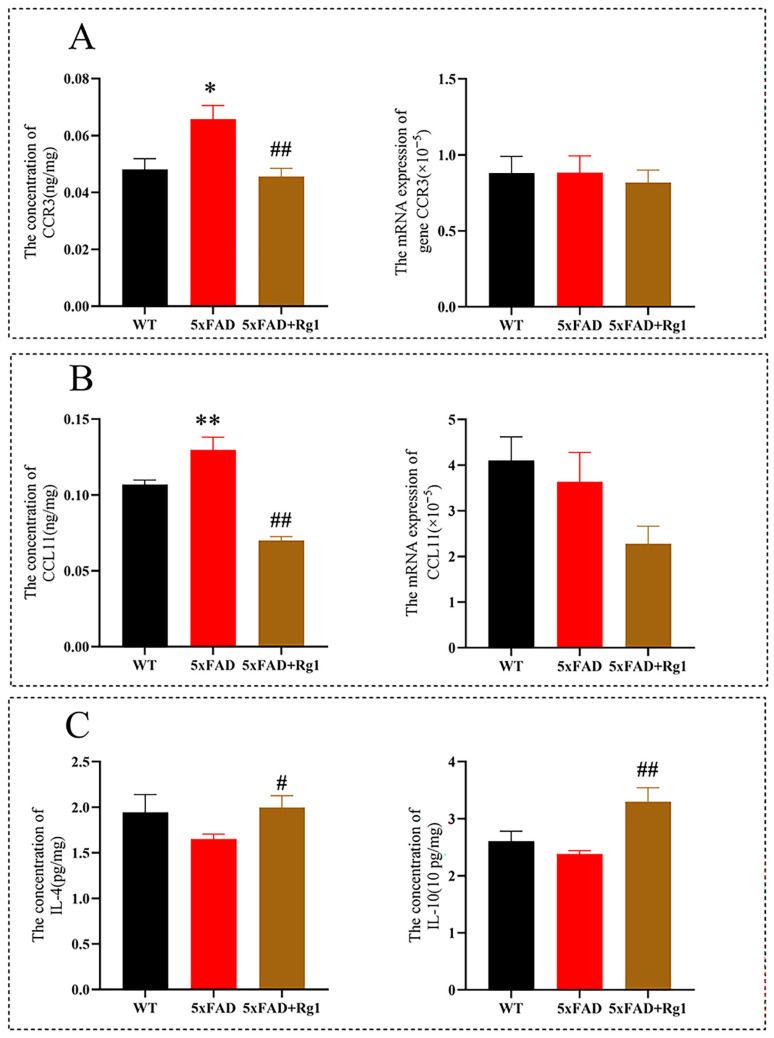
Rg1decreases the concentrations of CCR3 and CCL11 in the cortex of 5xFAD mice. (**A**) The level and mRNA expression of CCR3 in the cerebral cortex were detected by ELISA and RT-qPCR. (**B**) The level and mRNA expression of CCL11 in the cerebral cortex were detected by ELISA and RT-qPCR. (**C**) The concentrations of IL-4 and IL-10 in the hippocampus were detected via multiplex bead analysis. Mean ± SEM; *n* = 8. ** *p* < 0.01, * *p* < 0.05, vs. WT, Student’s *t*-test; ## *p* < 0.01, # *p* < 0.05, vs. 5xFAD, Student’s *t*-test.

The protein levels of CCR3 (Figure 3A; *p* < 0.05) and CCL11 (Figure 3B; *p* < 0.01) were markedly elevated in 5xFAD mice; however, treatment with Rg1 effectively attenuated these increases (Figure 3A,B; *p* < 0.01). In contrast, mRNA expression levels for both CCR3 and CCL11 remained unchanged across the experimental groups (Figure 3A,B). Furthermore, Rg1 administration significantly upregulated the concentrations of Interleukin-4 (IL-4) and Interleukin-10 (IL-10) compared to the untreated 5xFAD group (Figure 3C; *p* < 0.05 and *p* < 0.01, respectively).

### 2.4. Rg1 Directly Binds to CCR3 and Inhibits CCR3 Activity

**Figure 4:**
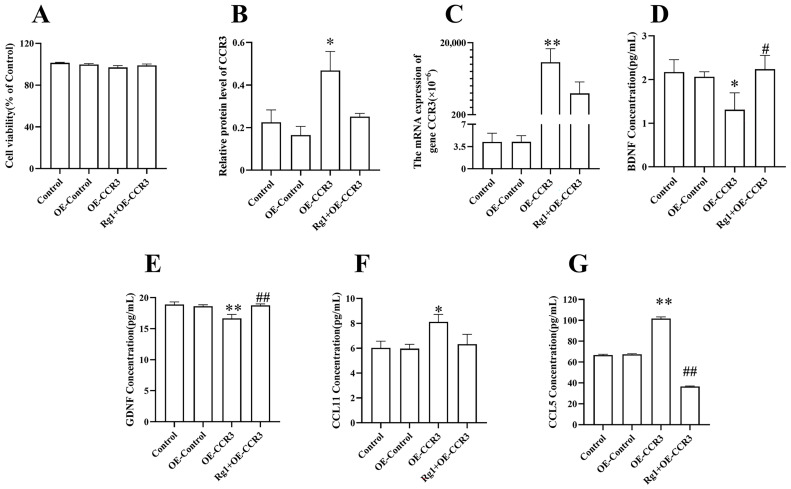
Rg1 performs a neuroprotective role by increasing neurotrophin and reducing chemokines in BV2 cells overexpressing CCR3. (**A**) Cell viability in the Cell Counting Kit-8 assay. (**B**) The protein expression of CCR3. (**C**) CCR3 mRNA expression. The concentrations of (**D**) CCL11, (**E**) CCL5, (**F**) BDNF and (**G**) GDNF. The control consisted of normal BV2 cells, OE-Control consisted of BV2 cells with LVCON335 overexpression, OE-CCR3 consisted of BV2 cells with Lv-CCR3-GFP overexpression and Rg1 + OE-CCR3 consisted of BV2 cells with Lv-CCR3-GFP overexpression after Rg1 treatment. Mean ± SEM; *n* = 3. ** *p* < 0.01, * *p* < 0.05, vs. OE-Control, one-way ANOVA followed by Dunnett’s multiple comparison test; ## *p* < 0.01, # *p* < 0.05, vs. OE-CCR3, Student’s *t*-test.

BV2 cells treated with or without a Lentivirus (Lv)–CCR3–GFP-overexpressing (OE-CCR3) vector or Rg1 had no significant difference in viability (Figure 4A). The concentrations and expression levels of CCR3 (Figure 4B,C and Figure S1; *p* < 0.01), as well as those of CCL11 and CCL5 (Figure 4D,E; *p* < 0.05, *p* < 0.01, respectively), in the OE-CCR3 group of BV2 cells increased. The concentrations of Brain-derived neurotrophic factor (BDNF) and Glial cell-derived neurotrophic factor (GDNF) (Figure 4F,G; *p* < 0.05; *p* < 0.01, respectively) were significantly reduced in the OE-CCR3 group of BV2 cells, but these levels were restored upon Rg1 treatment. A significant decline in CCL5 concentrations was observed following Rg1 intervention (Figure 4E; *p* < 0.01).

**Figure 5:**
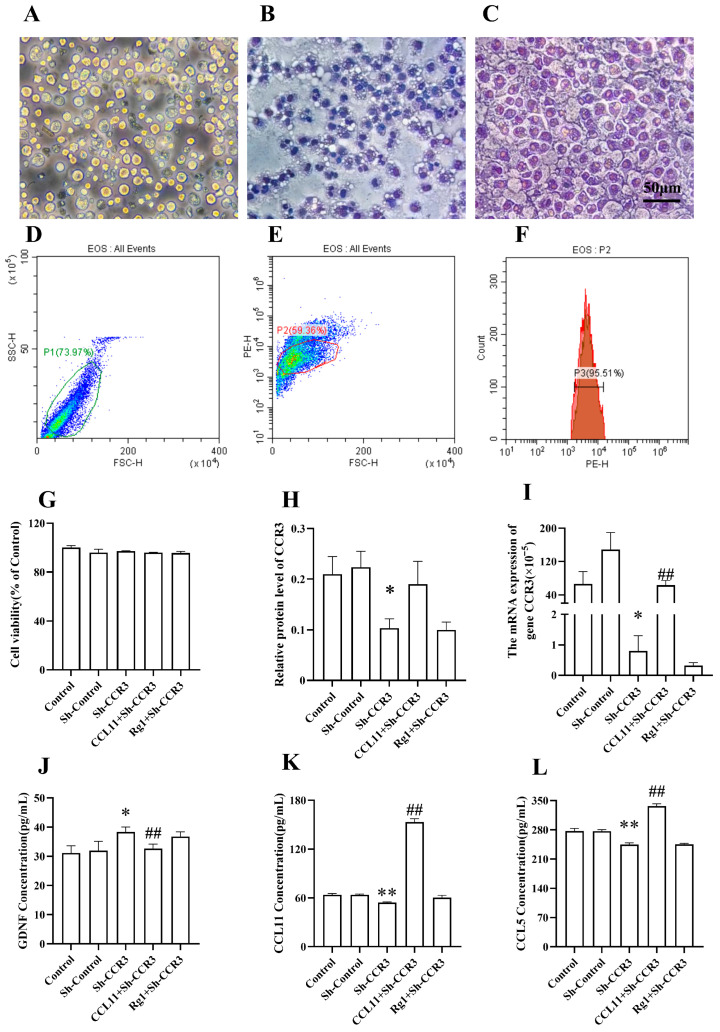
Rg1 had no significant influence on altering neurotrophin or chemokine levels in primary mouse eosinophils treated with shRNA targeting CCR3. (**A**–**C**) Mature primary EOSs and EOSs identified by Wright’s staining (scale bar = 50 μm, 400× magnification). (**D**–**F**) EOSs identified by flow cytometry. (**G**) Cell viability in the Cell Counting Kit-8 assay. (**H**) The protein expression of CCR3. (**I**) CCR3 mRNA expression. (**J**–**L**) The concentrations of CCL11, CCL5 and GDNF. The control consists of normal EOS cells, Sh-Control consists of EOS cells with LVCON313 transfection, Sh-CCR3 consists of EOS cells with Lv-ShRNA-CCR3 transfection and CCL11/Rg1 + Sh-CCR3 consists of BV2 cells with Lv-ShRNA-CCR3 transfection after CCL11 or Rg1 treatment. Mean ± SEM; *n* = 3. ** *p* < 0.01, * *p* < 0.05, vs. Sh-Control, one-way ANOVA followed by Dunnett’s multiple comparison test; ## *p* < 0.01, vs. Sh-CCR3, one-way ANOVA followed by Dunnett’s multiple comparison test.

EOSs induced from bone marrow were observed to grow in suspension and were round-shaped, with some cells growing in clusters; the cytoplasm contained eosinophilic granules (Figure 5A–C). The expression rate of Wright’s stain and flow cytometry (Siglec-F) on the primary EOS cell membrane was detected by flow cytometry and was 95.51% (mean ± SEM from at least three replicates), indicating the high purity of the primary EOSs (Figure 5D–F).

Neither Lv-short hairpin RNA (ShRNA)-CCR3 transfection nor Rg1 treatment induced significant changes in cell viability, indicating no cytotoxic effects on EOSs (Figure 5G). Both the concentrations and expression levels of CCR3 (Figure 5H,I and Figure S2; *p* < 0.05) in the Sh-CCR3 group were decreased compared with those in the Sh-Control group, as were those of CCL11 and CCL5 (Figure 5J,K; *p* < 0.01), whereas the concentrations of GDNF (Figure 5L; *p* < 0.05) were greater. However, these changes were reversed upon CCL11 treatment (Figure 5G–L). No differences were found in SH-CCR3-EOS cells before and after treatment with Rg1.

**Figure 6:**
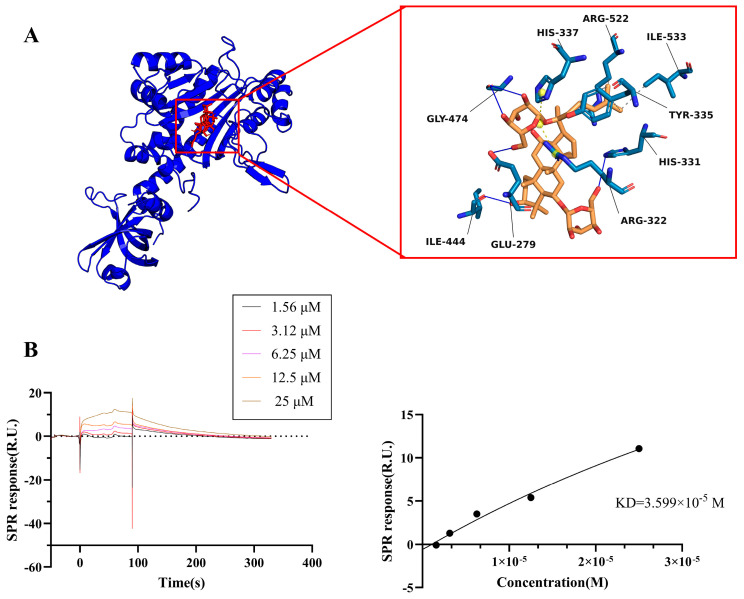
Rg1 directly binds to CCR3. (**A**) The most likely predicted binding sites of Rg1 in CCR3. (**B**) An SPR analysis of the binding of Rg1 to CCR3 (Kd = 3.599 × 10^−5^ mol/L).

Rg1 has a strong binding affinity for the 8H53 structure of CCR3, with a calculated binding energy of −8.77 kcal/mol according to the molecular docking results. Docking models between Rg1 and the 8H53 structure of CCR3 are illustrated (Figure 6A). To further substantiate the direct targeting of CCR3 by Rg1, surface plasmon resonance (SPR) was used, and the Kd of Rg1 binding to CCR3 was 3.599 × 10^−5^ mol/L (Figure 6B).

The binding affinity of Rg1 to CCR3 was found to be moderate. However, the functional assays in both in vitro and in vivo models revealed significant reductions in CCR3 and CCL11 levels following Rg1 treatment. Additionally, Rg1 treatment led to the restoration of neurotrophic factors, such as BDNF and GDNF. These findings suggest that CCR3 is a relevant target for the neuroprotective effects of Rg1. The results were further supported by complementary biochemical data, which strengthen the evidence for CCR3 inhibition as a key mechanism underlying the therapeutic effects of Rg1 in AD.

## 3. Discussion

In recent years, increasing evidence has suggested that chemokine signaling, particularly the CCR3/CCL11 axis, plays an important role in the regulation of microglial activation, eosinophil recruitment and neuroinflammatory progression [31,32]. The present study revealed that Rg1 significantly ameliorated cognitive deficits and Aβ burden in 5xFAD mice, accompanied by the suppression of CCR3/CCL11 protein expression and the restoration of neurotrophic factors such as BDNF and GDNF. This study expands our understanding of the molecular basis of Ginsenoside Rg1-mediated neuroprotection by identifying C-C motif chemokine receptor 3 as a critical therapeutic target.

A growing body of preclinical evidence indicates that Rg1 improves cognitive function in AD through a multitarget approach, which includes its antioxidant activity [29] and the ability to regulate nerve function [33]. However, the specific upstream targets associating Rg1 with neuroinflammation control remain largely undefined. The results in the present study reveal that Rg1 not only decreased CCR3 and CCL11 protein levels in both in vivo and in vitro models but also directly interacted with CCR3, as supported by molecular docking (binding energy of −8.77 kcal/mol) and SPR (Kd = 3.599 × 10^−5^ M). Although the observed binding affinity falls within a moderate range, the combined pharmacological and biophysical evidence suggests that CCR3 could be a direct or functionally relevant binding site for Rg1.

Previous studies have extensively documented the neuroprotective effects of various ginsenosides in AD models. These effects are often attributed to broad regulatory mechanisms, such as the modulation of antioxidant pathways, inhibition of neuroinflammation via nuclear factor kappa-B signaling or regulation of mitochondrial function [24,28]. However, the direct upstream molecular targets initiating these cascades have remained largely elusive. Unlike these general mechanisms, the present study uniquely identifies the chemokine receptor CCR3 as a specific direct target of Rg1. By demonstrating that Rg1 binds to and inhibits CCR3, the present study can provide a precise molecular explanation for its ability to modulate the immune microenvironment and restore neurotrophic support, distinguishing the present findings from the more generalized anti-inflammatory effects reported for other ginsenosides.

A considerable reduction in CCR3 and CCL11 protein expression without corresponding mRNA changes was observed, implying that Rg1 may regulate CCR3 post-transcriptionally. Possible mechanisms include the modulation of protein stability, translation efficiency or degradation pathways such as ubiquitination or lysosomal clearance. Future studies using proteasomal or lysosomal inhibitors will be required to clarify this mechanism. Regarding binding affinity, the value of M measured by SPR falls within the micromolar range. While this affinity is moderate compared with synthetic inhibitors, it is characteristic of the gentle modulatory effects often observed in natural products. Although the blood–brain barrier limits the instantaneous concentration of Rg1 in the brain, raising questions in regard to receptor occupancy, the findings in the present study suggest that the therapeutic effects may not require complete receptor blockade. Instead, the transient interaction between Rg1 and CCR3 may be sufficient to trigger sustained downstream signaling cascades, such as the restoration of BDNF and GDNF, which outlast the physical presence of the drug. Additionally, long-term administration may facilitate local accumulation in neuroinflammatory microenvironments. Therefore, while detailed pharmacokinetic studies and the direct quantification of Rg1-CCR3 occupancy in the brain are warranted in future investigations to fully establish physiological relevance, the significant functional recovery observed in this present study supports the validity of this target.

These cellular results substantiate the hypothesis that Rg1 exerts its neuroprotective effects via the CCR3 signaling pathway. In BV2 cells, CCR3 overexpression enhanced CCL11 and inflammatory cytokine production, which was markedly suppressed by Rg1 treatment. Conversely, in EOSs with CCR3 knockdown, Rg1 showed limited additional inhibitory effect, suggesting that CCR3 may be a necessary component of the anti-inflammatory action of Rg1. These findings corroborate the existing literature that underscores the role of CCR3 signaling in promoting neuroinflammatory processes and cognitive deterioration. The modulation of neurotrophic factors (BDNF and GDNF) further supports the notion that CCR3 inhibition by Rg1 may indirectly promote neuronal survival and synaptic function. It is worth noting that while Rg1 significantly improved cognitive function and reduced Aβ_1–40_ levels, it did not significantly lower the levels of the more neurotoxic Aβ_1–42_ isoform. This suggests that the therapeutic benefits of Rg1 may not rely solely on the clearance of amyloid plaques. Instead, the findings in the present study highlight the key role of CCR3 inhibition. By suppressing CCR3 activity, Rg1 attenuated neuroinflammation and restored the levels of neurotrophic factors (BDNF and GDNF). These neuroprotective mechanisms likely enhance neuronal resilience against Aβ-induced toxicity, thereby improving cognitive performance even in the presence of Aβ_1–42._

The present study revealed that CCR3 is a previously unrecognized target involved in the neuroprotective effects of Rg1. Through the inhibition of CCR3/CCL11 signaling, Rg1 may attenuate neuroinflammation, reduce Aβ accumulation and restore neurotrophic support, thereby improving cognitive performance in AD models. However, it is important to note that CCR3 is expressed not only in the brain but also in various peripheral immune cells, including EOSs, which are involved in systemic inflammation. In AD, peripheral immune activation, including eosinophil recruitment, contributes to the progression of neuroinflammation. Therefore, we hypothesize that Rg1 may exert its therapeutic effects not only by modulating CCR3 in the brain but also by influencing the activity of CCR3 in peripheral immune cells. This dual effect could help alleviate both central and systemic inflammation, thus providing a broader therapeutic benefit in AD. This systemic action of Rg1 underscores the potential for its peripheral pharmacological activity, which warrants further investigation, particularly regarding its pharmacokinetics and brain availability.

### Limitations and Future Perspectives

Several limitations of this study merit discussion. First, while SPR and docking analyses provide biophysical support for Rg1-CCR3 interaction, additional experiments using CCR3 antagonists, knockdown or knockout models in vivo are needed to confirm causal dependency. Second, the exact cellular sources and spatial distribution of CCR3 downregulation were not determined; immunohistochemical co-localization with microglia, astrocytes or vascular cells will be essential for clarifying the cellular targets. Third, Rg1 is a saponin with limited blood–brain barrier permeability; therefore, detailed pharmacokinetic analyses are required to evaluate its CNS bioavailability. These aspects will be key for translating the present findings into potential therapeutic applications.

To address these constraints, future research should prioritize several directions. Subsequent studies will incorporate the OFT to validate basal locomotor activity and utilize CCR3 knockout models to confirm the causal dependency of the CCR3/CCL11 axis. Furthermore, comprehensive pharmacokinetic studies are required to determine the brain bioavailability of Ginsenoside Rg1. Finally, while our models provide robust evidence, further clinical evaluations are essential to assess the efficacy and safety of Ginsenoside Rg1 in human Alzheimer’s disease patients, potentially exploring synergistic effects with other therapeutic agents for a more effective multitarget approach.

## 4. Materials and Methods

### 4.1. Reagents and Materials

Rg1 (CAS no. 22427-39-0) was procured from Shanghai Shanghai Winherb Medical Science Co., Ltd. (Shanghai, China). The Lv gene transfer vectors encoding overexpressed mouse CCR3 [22] and Lv-ShRNA-CCR3 were both purchased from Shanghai GeneChem Co., Ltd. (Shanghai, China), along with their respective unloaded Lv vectors. Recombinant human CCR3 was purchased from Shanghai Meiji Biotechnology Co., Ltd. (Shanghai, China).

### 4.2. Cell Culture

BV2 cells obtained from Elabscience Bionovation Inc. (Wuhan, China) were transfected with Lv-CCR3-GFP-OE (OE-CCR3), while LVCON335 served as the control (OE-Control).

Primary mouse EOSs, which express CCR3 and are involved in peripheral immune responses, were isolated and induced via a previously reported method [34]. Given that CCR3 is also expressed on peripheral immune cells such as EOSs, it is likely that Rg1 exerts its therapeutic effects through the modulation of both central and peripheral CCR3 activity. The process involved the extraction of bone marrow from the femurs and tibias of 10 C57BL/6J mice (*n* = 8; aged 6–8 weeks), followed by digestion with red blood cell lysis buffer (cat. no. R1010; Beijing Solarbio Science & Technology Co., Ltd. (Beijing, China)). The cells were then centrifuged and cultured in Stem Cell Factor and Fms-Related Tyrosine Kinase 3 Ligands (cat. nos. FLT3-L, 149-09CC-13 and 111-01CC-11; Proteintech Group, Inc. (Rosemont, IL, USA)), followed by the addition of the IL-5 protein (cat. no. 102303; MedChemExpress (Monmouth Junction, NJ, USA)). These cells were then identified by Siglec-F. The transfection of EOS cells was performed via the Lv-short hairpin RNA of CCR3 (Sh-CCR3, target sequence: CCATGCTCTAAGAATGAATAT), while LVCON313 served as the control (Sh-Control (Shanghai, China)).

Transfection efficiency was monitored using fluorescence microscopy. To establish the optimal Rg1 concentration, cell viability was evaluated via the Cell Counting Kit-8 (CCK-8) assay. Prior to transfection, BV2 and EOS cells were exposed to 50 μM Rg1 for 2 h, while an equivalent volume of DMSO served as the vehicle control.

This experiment was approved by the Experimental Animal Welfare Ethics Review Committee of Shandong University of Traditional Chinese Medicine (approval no. SDUTCM20230703223; approval date: 23 August 2023)). The Animal Research: Reporting in vivo Experiments (ARRIVE) guidelines were followed.

### 4.3. Western Blotting

Cells were lysed in a buffer containing a protease inhibitor cocktail (cat. no. GRF101; Ipsen Pharma, Paris, France). Protein lysates were separated via 5% or 7.5% Tris–Glycine SDS-PAGE and subsequently transferred to PVDF membranes. Following blocking with 5% BSA, membranes were incubated overnight at 4 °C with primary antibodies against CCR3 (1:500; cat. no. ab32512; Abcam, Cambridge, UK) and Glyceraldehyde-3-phosphate dehydrogenase (GAPDH) (1:500; cat. no. 60004-1-Ig; Proteintech, Rosemont, IL, USA). After washing, membranes were probed with an HRP-conjugated secondary goat anti-rabbit antibody (1:10,000; cat. no. EF0002; Shandong Sparkjade Scientific Instruments Co., Ltd. (Qingdao, China)) for 1 h at room temperature. Protein bands were visualized using the Omni-ECL Femto Light Chemiluminescence Kit (cat. no. SQ201; Ipsen Pharma (Paris, France)) on a Tanon-4800Multi imaging system. Quantification was conducted using ImageJ software (v1.54; NIH (Bethesda, MD, USA)), with GAPDH serving as the loading control.

### 4.4. Animals

5xFAD mice were used for the following experiments in the present study [35]. As a control group, age-matched WT mice were used. Male mice were bred and raised for 20 weeks and then genotyped (Table 1). Mice were housed in a specific pathogen-free facility under controlled conditions: a temperature of 22 ± 2 °C, relative humidity of 55 ± 5% and a 12 h light/dark cycle. The mice were housed in groups of 4–5 per cage with ad libitum access to standard rodent chow and water. This experiment was approved by the Experimental Animal Welfare Ethics Review Committee of Shandong University of Traditional Chinese Medicine (approval no. SDUTCM20230703223; approval date: 23 August 2023). The ARRIVE guidelines were followed.

### 4.5. Drug Administration

WT and 5xFAD mice (body weight, 25–30 g) were randomly divided by body weight. Each group had 8 mice: Group 1, WT mice; Group 2, 5xFAD mice; Group 3, 5xFAD + Rg1. Group 1 and Group 2 mice were given 0.5% CMC-Na. Group 3 mice were given 10 mg/kg/d Rg1. Rg1 was dissolved in 0.5% CMC-Na and administered orally (0.1 mL/10 g). The dosage of Rg1 has been documented in previous studies [29,36]. The experiment duration was 63 days. Animal health and behavior were monitored daily by trained animal care staff. Humane endpoints were strictly defined: mice exhibiting weight loss > 20%, severe lethargy, or inability to access food or water were to be euthanized immediately. Throughout this study, all animals remained in good health, and no unexpected deaths occurred. To minimize distress, mice were provided with environmental enrichment and handled gently. At the end of this experiment, animals were euthanized in a CO_2_ chamber (flow rate: 20% of chamber volume/min). Death was verified by the absence of respiration and heartbeat and confirmed by the absence of corneal reflex. The behavioral testing instruments and software (VisuTrack. 20230505) were supplied by Shanghai Xin Ruan Information Technology Co., Ltd. (Shanghai, China) (https://www.shxinruan.com/). Whole brains, cortical regions and plasma were collected.

### 4.6. Behavior Testing

#### 4.6.1. Y-Maze Test

The Y-maze test, which is used to evaluate short-term spatial memory in mice [31,37], was performed, and the specific experimental steps were consistent with those of a previous study [31]. The percentage of time spent in the novel arm and the number of entries into the novel arm during the test were recorded. Mice with fewer than 5 total arm entries were considered to have low exploratory motivation and were excluded from the analysis.

#### 4.6.2. OFT

General locomotor activity was assessed using the OFT, which also served as a habituation session prior to the NORT. The OFT was conducted one day before the NORT. The testing apparatus comprised a 50 × 50 × 50 cm square arena constructed from opaque material. Each mouse was placed in the center of the arena to explore freely for 10 min. Locomotor function was quantified by measuring the total distance traveled. To prevent olfactory interference, the arena was sanitized with 75% ethanol between each trial. This procedure ensured that the animals were familiarized with the environment, thereby minimizing anxiety-related interference during subsequent cognitive assessments.

#### 4.6.3. NORT

The NORT, which assesses object recognition memory [38,39], was performed according to the experimental steps reported in a previous study [31]. Exploration behavior was assessed using the Preference index (PI), which represents the relative time invested in investigating the novel object compared to the familiar object. Mice with a total exploration time of <10 s during the testing phase were excluded to ensure valid object interaction.

#### 4.6.4. Fear Conditioning Test

A fear conditioning test, which involves evaluating fear memory ability [40,41], was performed, and the specific experimental steps were consistent with those of a previous study [31]. During training, mice explored the chamber for 2 min, followed by 6 cycles of auditory stimulation (4 kHz, 80 dB, 15 s) paired with a foot shock (0.5 mA, 2 s), with 13 s intervals. Twenty-four hours later, mice were returned to the chamber for testing. After 2 min of free exploration, they were exposed to 6 cycles of the auditory cue (15 s) followed by a 15 s silence period. Freezing behavior was recorded to quantify fear memory. The freezing time during the training and testing phases was recorded. Mice exhibiting high baseline freezing (>20%) during the training phase or those defined as statistical outliers (±2 SEM from the mean) were excluded.

### 4.7. Enzyme-Linked Immunosorbent Assay (ELISA) and Multiplex Bead Analysis

Both cultured cells and mouse brain tissue were homogenized and centrifuged, and the total protein was quantified. The cortical concentrations of Aβ_1–40_, Aβ_1–42_ and CCR3 were measured. Cellular and cortical CCL11 concentrations, as well as GDNF, BDNF and CCL5 concentrations, were quantified in the cells. Multiplex bead analysis was used to analyze the hippocampal samples. The levels of IL-4, IL-10 and other cytokines were detected.

### 4.8. RNA Extraction and Reverse Transcription Quantitative Polymerase Chain Reaction (RT-qPCR)

Total RNA was extracted from both cells and the mouse hippocampus using steady pure universal RNA extraction Kit (cat. no. AG21017; Accrute Biotech Co., Ltd. (East Windsor, NJ, USA); https://agbio.com.cn/) to determine mRNA expression. The reverse transcription of RNA into cDNA was performed according to the manufacturer’s protocol for the Evo M-MLV RT Mix Kit with gDNA Clean for qPCR (cat. no. AG11728; Accrute Biotech Co., Ltd.). qPCR was performed with SYBR Green Premix Pro Taq HS qPCR Kit (cat. no. AG11718; Rox Plus; Accrute Biotech Co., Ltd.) using a Gentier 96R Real-Time PCR System (Xi’an Tianlong Science and Technology Co., Ltd. (Xi’an, China); https://www.medtl.com/), and the primers were synthesized by the Sangon Biotech Co., Ltd. (Shanghai, China). The PCR procedure was conducted as follows: 95 °C for 2 min, followed by 40 cycles of 95 °C for 10 s, followed by 62 °C for 1 min. Target gene expression was normalized to that of β-actin. This value was calculated via the 2^−ΔΔCq^ method [42]. The sequences of primers are listed in Table 2.

### 4.9. Molecular Docking and SPR

AutoDock Vina 1.5.7 was employed to investigate the molecular interaction between CCR3 and Rg1 [43]. The structure of human CCR3 (PDB ID, 8H53) was retrieved. The structural data for Rg1 were obtained from a database (PubChem, https://pubchem.ncbi.nlm.nih.gov/; accessed on 10 August 2024). The grid dimensions were set to 20.01 × 20.01 × 20.01 Å, with the center positioned at the active site pocket coordinates (31.948, 13.819, 42.079). The docking parameters were configured with 100 runs for exhaustiveness, a maximum of 50 modes (num_modes) and an energy range of 3 kcal/mol.

A Biacore T200 was used. Briefly, CCR3 was immobilized on a CM5 chip, and a series of gradient concentrations of Rg1 was diluted in running buffer as mobile phases.

### 4.10. Statistical Analysis

The data are presented as the means ± SEM. GraphPad Prism version 8.0.1 was utilized for data visualization and statistical analysis. An unpaired Student’s *t*-test was applied to compare two groups, whereas a one-way ANOVA, followed by Dunnett’s multiple comparison test, was used to assess the differences between multiple groups and a control. *p* < 0.05 was considered to indicate a statistically significant difference.

## 5. Conclusions

In summary, the present study identifies CCR3 as a previously unrecognized pharmacological target of Rg1. We provide multi-level evidence that Rg1 ameliorates cognitive deficits and amyloid-beta pathology in 5xFAD transgenic mice by inhibiting the CCR3/CCL11 axis, thereby suppressing neuroinflammation and restoring neurotrophic support, including BDNF and GDNF. These findings not only clarify the molecular basis underlying the neuroprotective effects of Rg1 but also highlight CCR3-mediated chemokine signaling as a promising therapeutic target in Alzheimer’s disease. However, further studies are required to elucidate the brain pharmacokinetics of Rg1, validate the causal role of CCR3 using genetic models, and determine its cell-specific mechanisms of action. In addition, future work should explore the long-term efficacy and safety of Rg1, as well as its translational potential in clinical settings for AD therapy.

## Figures and Tables

**Table 1:** The primers used for genotyping.

Name	Primer Sequences (5′-3′)
APP	Forward: GCCAACCAACCAGTGACCATCC
	Reverse: TCCGCATCAGCAGAATCCACATTG
β-actin	Forward: TTCCCGCGGTGTAGACACTC
	Reverse: TGGACAAAGACCCAGAGGCC

**Table 2:** The primer sequences for RT-qPCR.

Name	Primer Sequences (5′-3′)
CCR3	Forward: GCTTTGAGACCACACCCTATGAAT
	Reverse: AACCATCATGTTGCCCAGGAG
CCL11	Forward: ATTCCTGCTGCTCACGGTCAC
	Reverse: TGTAGCTCTTCAGTAGTGTGTTGGG
β-actin	Forward: TTCCCGCGGTGTAGACACTC
	Reverse: TGGACAAAGACCCAGAGGCC

## Data Availability

The original contributions presented in this study are included in the article. Further inquiries can be directed to the corresponding author.

## Supplementary Materials

The following supporting information can be downloaded at https://www.mdpi.com/article/10.3390/ph19050661/s1. Figure S1: (Related to Figure 4B). CCR3 and GAPDH protein levels in OE-CCR3 BV2 cells were detected using western blotting. The corresponding quantitative analysis (bar chart) is presented in Figure 4B. (A) Western Blot Analysis of CCR3 and GAPDH Protein Bands. (B) The complete uncropped western blot original images with marker bands of CCR3. (C) The complete uncropped western blot original images with marker bands of GAPDH. The effects of different single compounds were simultaneously tested on the CCR3 protein of OE-CCR3 BV2 cells to save time and resources. CCR3, C-C chemokine receptor type 3; OE, overexpression. Figure S2: (Related to Figure 5H). CCR3 and GAPDH protein levels in Sh-CCR3 EOS cells were detected using western blotting. The corresponding quantitative analysis (bar chart) is presented in Figure 5H. (A) Western Blot Analysis of CCR3 and GAPDH Protein Bands. (B) The complete uncropped western blot original images with marker bands of CCR3. (C) The complete uncropped western blot original images with marker bands of GAPDH. The effects of different single compounds were tested on the CCR3 protein of Sh-CCR3 EOS cells in order to save time and resources. Figure S3: Cell culture and identification images of BV2 cells. The original image of BV2 cells after lentivirus transfection observed under a bright-field microscope. (B) The original fluorescence image of BV2 cells after lv transfection observed under fluorescence microscopy. (C) The merged image.

## Abbreviations

The following abbreviations are used in this manuscript:

AD	Alzheimer’s disease
ANOVA	Analysis of variance
Aβ	Amyloid-beta
BDNF	Brain-derived neurotrophic factor
CCL5	C-C motif chemokine ligand 5
CCL11	C-C motif chemokine ligand 11
CCR3	C-C motif chemokine receptor 3
ELISA	Enzyme-linked immunosorbent assay
EOS	Eosinophil
IL-4	Interleukin 4
IL-10	Interleukin 10
GAPDH	Glyceraldehyde-3-phosphate dehydrogenase
GDNF	Glial cell-derived neurotrophic factor
Rg1	Ginsenoside Rg1
Lv	Lentivirus
NORT	Novel object recognition test
OE	Overexpression
OFT	Open field test
PI	Preference index
RT-qPCR	RNA extraction and reverse transcription quantitative polymerase chain reaction
Sh	Short hairpin
SPR	Surface plasmon resonance
WT	Wild-type C57BL/6J
