# Supplementary material for: Ginsenoside Rg1 Ameliorates the Learning and Memory Deficits of 5xFAD Mice by Inhibiting CCR3 Activity: Insights from In Vivo and In Vitro Investigations
Source: Pharmaceuticals (Basel). 2026 Apr 23;19(5):661. doi: 10.3390/ph19050661 (PMC13209778; doi:10.3390/ph19050661)
Supplement: Supplementary file 1 [file pharmaceuticals-19-00661-s001.zip › pharmaceuticals-4257367-supplementary.pdf]

Figure S1: CCR3 and GAPDH protein levels in OE-CCR3 BV2 cells were detected using western blotting.

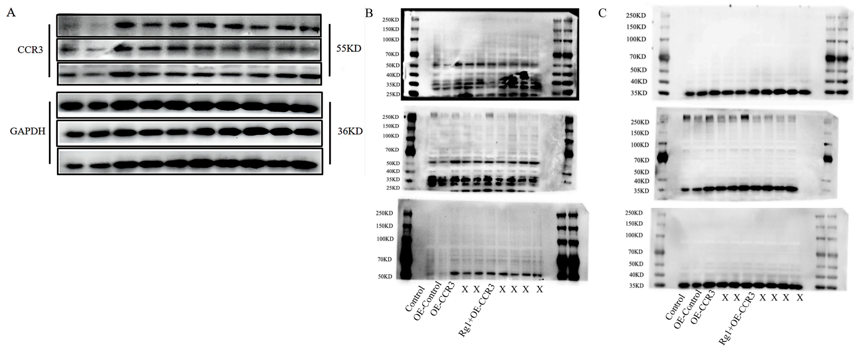

Figure S1. (Related to Fig. 4B). CCR3 and GAPDH protein levels in OE-CCR3 BV2 cells were detected using western blotting. The corresponding quantitative analysis (bar chart) is presented in Fig. 4B. (A) Western Blot Analysis of CCR3 and GAPDH Protein Bands. (B) The complete uncropped western blot original images with marker bands of CCR3. (C) The complete uncropped western blot original images with marker bands of GAPDH. The effects of different single compounds were simultaneously tested on the CCR3 protein of OE-CCR3 BV2 cells to save time and resources. CCR3, C-C chemokine receptor type 3; OE, overexpression.

Figure S2: CCR3 and GAPDH protein levels in Sh-CCR3 EOS cells were detected using western blotting.

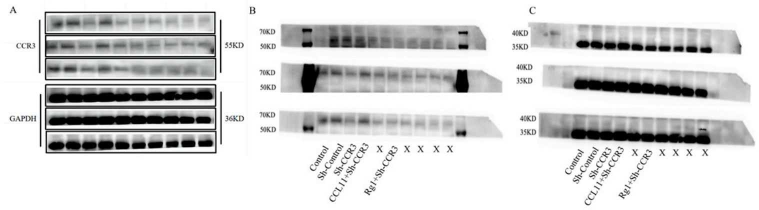

Figure S2. (Related to Fig. 5H). CCR3 and GAPDH protein levels in Sh-CCR3 EOS cells were detected using western blotting. The corresponding quantitative analysis (bar chart) is presented in Fig. 5H. (A) Western Blot Analysis of CCR3 and GAPDH Protein Bands. (B) The complete uncropped western blot original images with marker bands of CCR3. (C) The complete uncropped western blot original images with marker bands of GAPDH. The effects of different single compounds were tested on the CCR3 protein of Sh-CCR3 EOS cells in order to save time and resources.

Figure S3: Cell culture and identification images of BV2 cells.

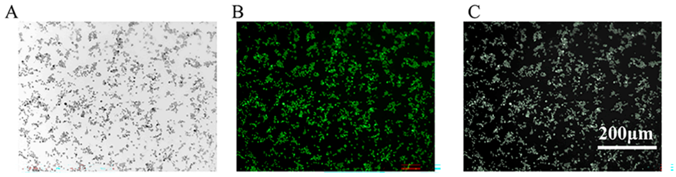

Figure S3. Cell culture and identification images of BV2 cells. The original image of BV2 cells after lentivirus transfection observed under a bright-field microscope. (B) The original fluorescence image of BV2 cells after lv transfection observed under fluorescence microscopy. (C) The merged image.
